# Valorization of Mushroom By-Products as a Source of Value-Added Compounds and Potential Applications

**DOI:** 10.3390/molecules25112672

**Published:** 2020-06-09

**Authors:** Filipa Antunes, Sara Marçal, Oludemi Taofiq, Alcina M. M. B. Morais, Ana Cristina Freitas, Isabel C. F. R. Ferreira, Manuela Pintado

**Affiliations:** 1 CBQF–Centro de Biotecnologia e Química Fina–Laboratório Associado, Escola Superior de Biotecnologia, Universidade Católica Portuguesa, Rua Diogo Botelho 1327, 4169-005 Porto, Portugal; fantunes@porto.ucp.pt (F.A.); sara.ad.marcal@gmail.com (S.M.); abmorais@porto.ucp.pt (A.M.M.B.M.); afreitas@porto.ucp.pt (A.C.F.); 2 Centro de Investigação de Montanha (CIMO), Instituto Politécnico de Bragança, Campus de Santa Apolónia, 5300-253 Bragança, Portugal; taofiq.oludemi@ipb.pt (O.T.); iferreira@ipb.pt (I.C.F.R.F.)

**Keywords:** mushroom by-products, valorization, bioactive compounds, applications

## Abstract

Nowadays, the food sector is highly concerned with environmental issues and foreseen to develop strategies to reduce waste and losses resulting from activities developed in the food system. An approach is to increment added value to the agro-industrial wastes, which might provide economic growth and environmental protection, contributing to a circular economy. Mushroom by-products represent a disposal problem, but they are also promising sources of important compounds, which may be used due to their functional and nutritional properties. Research has been developed in different fields to obtain value added solutions for the by-products generated during mushroom production and processing. Bioactive compounds have been obtained and applied in the development of nutraceutical and pharmaceutical formulations. Additionally, other applications have been explored and include animal feed, fertilizer, bioremediation, energy production, bio-based materials, cosmetics and cosmeceuticals. The main purpose of this review is to highlight the relevant composition of mushroom by-products and discuss their potential as a source of functional compounds and other applications. Future research needs to explore pilot and industrial scale extraction methods to understand the technological feasibility and the economic sustainability of the bioactive compounds extraction and valorization towards different applications.

## 1. Introduction

Edible mushrooms have long been consumed both as valuable protein and energy sources, and also to improve human health and longevity. They are considered healthy foods, being both low in calories and fat, and rich in proteins, minerals and dietary fiber [1,2]. The production and consumption of mushrooms is growing each year, given their nutritional advantages in addition to their flavor, increasingly appreciated by the world population [3]. In the mushroom production process, a large amount of by-products is generated, with a high environmental impact and management costs for the industry. By-products include caps, stipes, mushrooms that do not comply with commercial standards in terms of caliber, shape or size and spent mushroom substrate (SMS). SMS is composed of fungal mycelia, extracellular enzymes secreted from mushrooms for degradation of substances and unused lignocellulosic substrates [4]. These by-products have a high nutritional value and can be valued in several applications.

Nowadays, industrial ecology concepts, such as circular economy, are considered leading principles for innovation using waste as raw material for the development of new products and applications [5]. The large amount of waste generated by mushroom production represents a huge loss of valuable organic materials raising also serious management problems, both from the economic and environmental point of view.

Though a few reviews are available on SMS applications [6,7], no review is currently available on extraction of bioactive compounds from mushroom by-products and its potential applications in different fields, such as food, animal feed, fertilizer, bioremediation, energy production and bio-based materials. To the best of our knowledge, this is the first review on valorization of mushroom by-products, in different fields, resulting from the two mushroom production techniques (solid substrate fermentation only or including submerged liquid fermentation steps). It is well known that mushroom production is obtained from solid substrate fermentation, but the production process can include submerged liquid fermentation to produce liquid spawn for mushroom production. Therefore, this review aims to present and discuss the potential applications of mushroom by-products being focused on the extraction of bioactive compounds which could result in nutraceuticals with high economically value.

## 2. Mushroom Production

**Figure 1:**
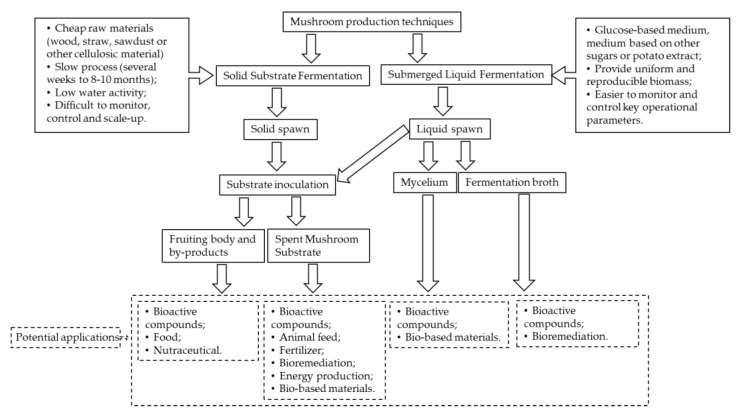
Schematic diagram of the mushroom production techniques, main products derived from production and potential applications. Adapted from [1,12].

Although thousands of mushrooms exist in the wild, only around 25 species are used extensively as food and few are commercially cultivated [8]. Amongst the different cultivated mushrooms, *Lentinula* is the major genus, contributing about 22% of the world’s cultivated mushrooms and *Pleurotus* constitutes about 19% of the world’s output [9]. *Agaricus* and *Flammulina* are responsible for 15% and 11% of the volume, respectively [9]. It has long been known that saprophytic mushrooms develop biomass through degrading cellulose and lignin by the action of specific enzymes at optimal conditions [10]. As already mentioned, mushroom production is comprised of two types of fermentation techniques: solid substrate fermentation only or including submerged liquid fermentation steps (Figure 1). Solid substrate fermentation is the chosen production method of whole mushrooms for food and nutraceutical purposes [11]. Submerged liquid fermentation is used in mycelium propagation for different applications, such as liquid spawn (substrate inoculated with mycelium used for propagation of mushroom) for fruiting body (or sporocarp) production on solid substrates, biomass production for food, dietary supplement and pharmaceutical applications and conversion of waste biomass and enzyme production [11]. In all cases the underlying principle in each approach is to use the mycelium in the active physiological state and of known purity. The main products derived from mushroom production, both solid substrate fermentation and submerged liquid fermentation and potential applications, are presented in Figure 1. The following topics describe the major steps involved in the production of mushrooms.

### 2.1. Solid Substrate Fermentation

Mushroom culture is a biotechnological process that enable to recycle lignocellulosic wastes, since these are converted to a food for human consumption and the SMS can be used in several applications [13]. Solid substrate fermentation is the most preferred mushroom production technique that involves supplying mushroom substrate to growers in polyethylene bags, with all ingredients including mushroom spawn. The fruiting body is then formed from spacious mycelium as a result of the fructification process and mushrooms are generated in several weeks [14]. As already pointed and discussed, the production of mushrooms generates a large amount of waste in the form of caps, stipes, mushrooms that do not comply with commercial standards and SMS. Depending on the size of the mushroom farm, the amount of mushroom stipes and poor quality mushrooms ranges from 5 to 20% of the production volume. Waste disposal creates environmental problems for producers due to the large volume and volatile degradation products [15]. The production of mushrooms results in significant residual material after cultivation—every kilogram of mushrooms produced results in 5 to 6 kg of by-product [16]. Traditionally, incineration has been applied for the final disposal of abandoned SMS, which could cause a series of environmental problems, including air pollution [17]. In addition, caps and stipes that are misshaped or do not meet the specifications set by retailers are discarded [18,19]. Thus, it is necessary to implement new techniques for the use of SMS as well as of misshaped caps and stipes in valuable applications. 

In the last decades, mushroom production on solid substrate fermentation only has been studied because it allows the use of a large variety of agro-industrial waste as a substrate. High yields of production are provided since it simulates the natural environment of mushroom development, and it uses low power consumption [20]. Substrates offered for mushroom production normally contain organic nitrogen sources and are low in free ammonium, since excess can inhibit growth or fruiting of mushroom [21]. Basically, solid substrate fermentation recycles lignocellulosic wastes from industry and agriculture into different applications, such as edible biomass, animal nutrition, bioremediation, functional compounds and drug development.

**Figure 2:**
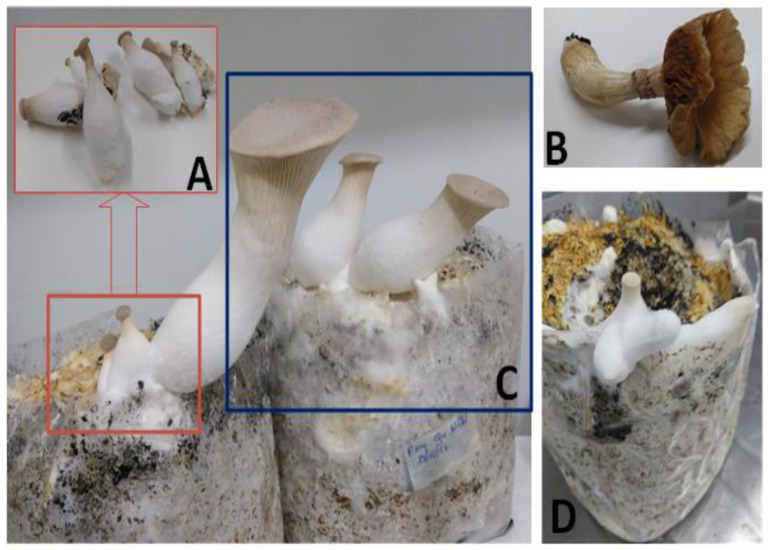
Mushroom by-products obtained by solid substrate fermentation only. (**A**) and (**B**) are mushroom production wastes (stipes and mushrooms that do not comply with commercial standards in terms of caliber, shape or size: 5–20% of the production weight); (**C**) by-products (surplus production: ≤ 5%); (**D**) spent mushroom substrate (>20% of the production weight). The photos were graciously supplied by Voz da Natureza.

The following sections describe the main by-products derived from mushroom production: fruiting bodies (stipes and caps), SMS from solid substrate fermentation (Figure 2) and mycelium from either solid substrate fermentation present in SMS or from submerged liquid fermentation.

#### 2.1.1. Fruiting Body

The main application of fruiting body from mushroom production is for food and medicinal uses. Several studies have focused on the characterization and extraction of bioactive compounds from different mushroom species, such as *Flammulina velutipes* [22], *Ganoderma lucidum* [23,24], *Laetiporus sulphureus* [25], *Pholiota nameko* [26], *Pleurotus eryngii* [27], *Poria cocos* [28], *Pleurotus ostreatus* [29] and *Schizophyllum commune* [30]. Nevertheless, stipes and mushrooms that do not comply with commercial standards and surplus production represent an interesting raw material for the extraction of value-added compounds, and there is a lack of research in this particular field. The stipe of shiitake mushroom (*Lentinula edodes*) has typically been discarded because of its tough texture [31]. However, shiitake stipes can be used as an alternative nitrogen source in alcoholic fermentation because of their high protein content [31]. In a recent study, the nutritional composition of *Agaricus bisporus* by-products (stipes and caps) powder was determined. Total carbohydrates including polysaccharides, such as glucans, mono- and disaccharides, sugar alcohols, glycogen and chitin, were 53.07 g/100 g Dry Matter (DM), which was found to be in the same range than those values reported in other edible mushroom species [18,32]. The variability in terms of composition could be dependent on the mushroom species and other parameters, such as environmental temperature and relative humidity during growth that may be produced or used during storage.

#### 2.1.2. Spent Mushroom Substrate

Spent mushroom substrate (SMS) is the main by-product in volume and weight of mushroom industry after the sporocarp has been removed from the culture and is a composted organic medium that results from the mushroom cultivating process. The term “spent mushroom compost” is used interchangeably with SMS describing the agro-residues and fungal mycelium left after harvesting the mushrooms. This waste is commonly made from renewable agricultural residues such as sawdust, sugarcane bagasse, oil palm fruit-free bunch, wheat straw-bedded horse manure, hay, poultry manure, ground corncobs, cottonseed meal, cocoa shells, gypsum among other substances [33]. There are two kinds of substrates for cultivation which varies according to mushrooms type: composted and pasteurized substrate [34]. *Agaricus* spp. are cultivated on compost substrate, typically on a straw base, amended with animal manures and gypsum. The materials initially undergo a two-phase composting process. *Pleurotus* spp. are cultivated on pasteurized substrate, usually wood sawdust and various plant fibers. The growing materials are sterilized, pasteurized or chemically treated to augment the selectivity of these materials for the oyster mushroom fungus [35]. Depending on mushroom species, each cultivation cycle varies for 1 to 2 months for *Agaricus bisporus* [36] or *Pleurotus ostreatus* [37] and 5 to 6 months for *Auricularia* spp. [38] from which the resulting SMS is disposed [7]. Tons of SMS are commercially produced worldwide every year, which is rich in natural polymers, such as chitosan, chitin, protein, cellulose and hemicelluloses [39]. SMS contain mycelium with high content of compounds such as trace elements (Fe, Ca, Zn and Mg), cellulose, hemicellulose, lignin, carbohydrate, crude protein and fat [40]. Due to the high nutritional value of SMS and the current environmental impact a more efficient use is urgent for resource recycling, environmental protection and sustainable development of mushroom industry [41]. Some of the researched possible solutions are bioremediation, animal feed, energy feedstock, crop production and enzyme recovery [3,7]. The use of SMS to obtain enzymes has the advantage of using a low cost product, adding value to wastes and producing high value products [7,42]. Several researches have studied that SMS polysaccharides present bioactivities, including antibacterial, antitumor, antioxidant and renoprotective [40,43,44,45].

### 2.2. Submerged Liquid Fermentation

Submerged liquid fermentation offers the possibility of high biomass production in a minor volume, shorter time with higher control of contamination. While solid substrate fermentation still remains the chosen method for mushroom production, there will be a continuous increase in the development of submerged liquid fermentation technology in order to produce more uniform and reproducible biomass of medicinal fungi [46,47]. Submerged liquid fermentation have been proven to be interesting for valuable medicinal products or for enzyme production, because of simple downstream processing [11,48]. A commercial scale depends on a better understanding of the bioprocess and engineering aspects for the production of mushroom mycelium and metabolites [20]. The main biotechnological steps to achieve highly nutritive mycelial biomass by controlled submerged liquid fermentation are: (i) preparation of culture medium, (ii) steam sterilization of the bioreactor culture vessel, (iii) aseptic inoculation of sterilized culture media with the pure cultures of selected mushroom strains, (iv) running the submerged cultivation cycles under controlled conditions of temperature, aeration and pH and (v) collecting, washing and filtering the obtained fungal pellets [49].

#### 2.2.1. Mycelium

Submerged culture of mushrooms is a promising alternative for efficient production of mycelium and metabolites and it has received increasing attention around the world [10]. The components of the culture media, including the concentration and type of carbon and nitrogen sources, initial pH, inoculum volume and agitation speed are main factors that significantly affect biomass production [50]. Instead of using fruiting bodies as source of bioactive compounds, the use of mycelia grown in submerged liquid culture, guarantee a more reliable product with higher level of control of its composition. Even for mushroom production, the production of liquid spawn offers some advantages including low contamination, high efficiency, low cost and production stability [51]. Submerged liquid fermentation provides an opportunity to optimize yields and enables to manipulate the chemical and physical properties of the metabolites improving their bioactivities [1]. Furthermore, as most metabolites are synthesized extracellularly, their recovery is much simpler too. However, the eventual exploitation of submerged liquid fermentation or solid substrate fermentation will be determined by their productivity and biological effectiveness. For example, chemical and biological properties of the polysaccharide produced in submerged liquid culture are quite different from those synthesized by the fruiting body and, in some cases, no polysaccharide is produced or they lack any biological activity due to structural differences [52]. It must be stated that the polysaccharide structure in cultured mycelia may depend on the composition of the nutrient medium used for cultivation [53]. In addition, polysaccharides extracted from fruiting bodies or from cultured mycelium of the same species are also dependent on the method of fractionation used but, in general, the total content of polysaccharides is higher in fruiting bodies [54].

#### 2.2.2. Fermentation Broth

The fermentation broth from submerged liquid culture is rich in extracellular polysaccharides with bioactivity [55]. In view of the importance of extracellular polysaccharides, many attempts have been made to obtain these compounds from submerged liquid fermentation. The main roles of extracellular polysaccharides include fungi adhesion to the substrate, immobilization of extracellular enzymes, prevention of hyphal dehydration, storage of excess nutrients and participation in lignin degradation [46]. Mycelium contains polysaccharides as glucans (homopolymers of glucose), and produces these often in large amounts extracellularly, under appropriate conditions [1]. Bioactive compounds released for the fermentation broth become interesting for several applications such as animal feed [56] and functional food [57]. Medium constituents showed effects on composition, structure and productivity of extracellular polysaccharides [58]. Additionally, the optimal submerged culture conditions for maximum extracellular polysaccharides production depends strongly on the species and strains used [59].

## 3. Bioactive Compounds and Extraction Methods

Bioactive compounds are constituents that occur in variable quantities in foods [60] and are capable of modulating metabolic processes resulting in the promotion of better health [61]. Bioactive compounds have diverse structures and functionalities, having enormous potential for the production of nutraceuticals, functional foods and food additives [62]. The bioactive compounds of mushrooms include polysaccharides (e.g., beta-glucans), proteins, fats, minerals, glycosides, alkaloids, volatile oils, tocopherols, phenolics, flavonoids, carotenoids, folates, ascorbic acid, enzymes and organic acids [63]. The cell walls of mushrooms contain chitin, hemicelluloses, glucans, mannans and especially branched non-cellulosic beta-glucans, which are thought to have beneficial health properties [1]. The concentration and efficacy of the bioactive compounds are variable and depend on the mushroom species, type of substrate, fruiting conditions, stage of development, storage conditions and cooking procedures [64]. At present, 80–85% of all medicinal mushroom products are derived from fruiting bodies, which have been either commercially farmed or collected from the wild. Only 15% of all products are based on extracts from mycelia [46,65]. Despite the discovery of many medicinal mushrooms and the identification of their bioactive polysaccharides, there are only a handful of functional food products where these polysaccharides are used. This is probably because there are several concerns regarding the application of these important molecules in final food products, such as the diversity of the biopolymers, the unstable quantity, quality and availability of these mushroom polysaccharides, the impact of the purification process and food processing on the bioactivity of the biopolymers and their production costs [66]. Additionally, safety issues and regulation are essential for considering the marketing of functional food products [67]. An example of this point is the limited practical applications of ergosterol and vitamin D_2_ from mushroom by-products, which requires further work to achieve food and pharmaceutical industries applications [68].

The majority of the studies concerning the mushrooms potential bioactivity are conducted with samples of fruiting bodies [25,28,69,70,71,72,73,74,75,76,77,78]. Nevertheless, the by-products generated from mushroom production also represent a good source of valuable compounds. These bioactive compounds could originate from solid substrate fermentation (caps and stipes from fruiting bodies, mycelium from SMS and SMS) or from submerged liquid fermentation (fermentation broth and surplus mycelium). 

**Figure 3:**
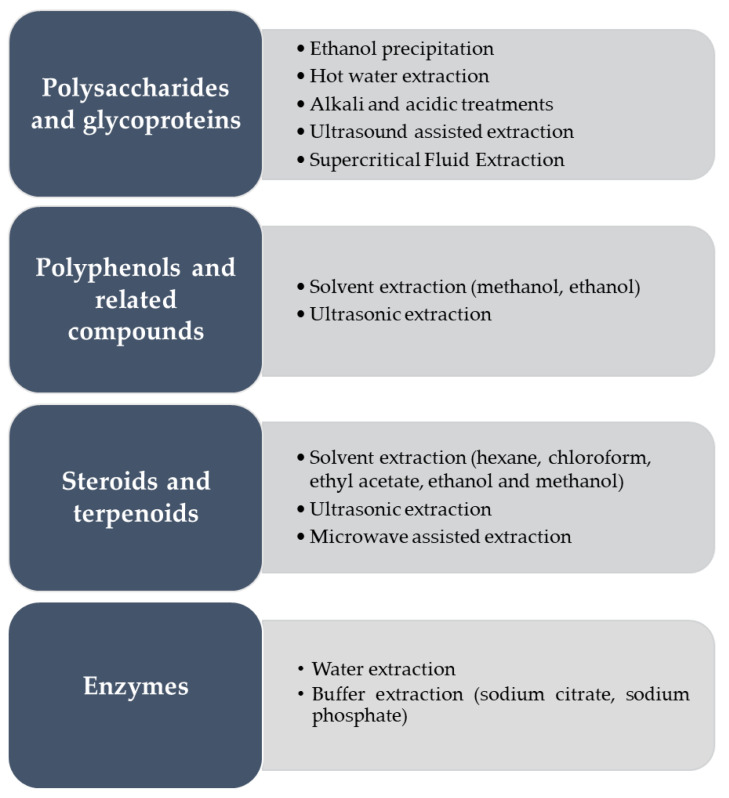
Main bioactive compounds in mushroom by-products and extraction methods involved in the extraction of bioactive compounds from mushroom by-products.

The following section describes the main bioactive compounds and extraction methods from mushroom by-products (Figure 3) obtained either by solid substrate fermentation only or including submerged liquid fermentation in the production steps.

### 3.1. Polysaccharides and Glycoproteins

Recently, increasing attention has been paid to polysaccharides as an important class of bioactive natural products [79]. Numerous researches have demonstrated bioactive properties of natural polysaccharides, which lead to the application of polysaccharides in the treatment and prevention of diseases [79]. Hot water extraction is a very interesting method for polysaccharide extraction from mushroom by-products, because of its safety and environment friendly compatibility [80]. High temperature can accelerate the dissolution of polysaccharide from cell walls [78]. Acidic, saline and diluted alkali solutions are also used to extract the polysaccharides in the basic rule of breaking cell walls from the outer to inner layers with mild-to-strong conditions [78]. In general, the traditional procedures for extraction are carried out as follows: powders of raw materials are firstly defatted with organic solvents to eliminate low molecular substances, successively extracted by hot water, saline and diluted alkali solutions at different temperatures [81,82]. Furthermore, other methods such as microwave, ultrasonic, ultrasonic/microwave and enzymatic method are also used, which could promote the breakage of cell wall and increase the yield of polysaccharides [26,70,78,83]. The main studies reported in the literature on extraction methods of polysaccharides from by-products are summarized in Table 1.

Chitin and chitosan have great applicability in various fields because they are non-toxic, biodegradable, biocompatible and have antimicrobial effects [105]. Mushrooms are considered an alternative source of chitin and chitosan because their cellular wall has a high content of chitin, which may be transformed into chitosan through a deacetylation reaction [105]. Wu et al. [15] performed chitin extraction from *A. bisporus* stipes. They found that chitin content reached 19% dry weight. The crude chitin from *A. bisporus* stipes was composed of a chitin-glucan complex [15]. Other recent studies focused on chitin extraction from mushroom by-products demonstrate its potential to be used in several applications. For example, *A. bisporus* stipes from mushroom by-product were treated with UV-B light to rapidly increase vitamin D2 content and chitin was recovered from this by-product and converted into chitosan by *N*-deacetylation. This study demonstrated that mushroom by-product could be used to develop chitosan films fortified with vitamin D2 [85]. Poverenov et al. [84] in turn found that *A. bisporus* stipe gave rise to a higher chitosan yield than caps (176 vs. 105 mg/g). This study demonstrated that mushroom by-products can be used for the production of chitosan-based edible coatings in fresh-cut melons due to fruit firmness enhancement, off-flavors inhibition and antimicrobial activity.

Three extractable polysaccharides including acidic residue polysaccharide, alkalic residue polysaccharide and enzymatic residue polysaccharide were extracted from SMS of *F. velutipes* and their antioxidant and renoprotective effects on streptozotocin-induced mice were investigated. The results demonstrated that enzymatic polysaccharide extracted from SMS of *F. velutipes* possessed potent antioxidant activity and could be used as a promising therapeutic agent for inhibiting the progression of diabetic nephropathy [40]. A novel water-soluble polysaccharide from *P. eryngii* mycelium from SMS was isolated and further purified by diethylaminoethyl (DEAE) cellulose-52 chromatography and Sephadex G-100 size-exclusion chromatography to yield three purified polysaccharides extracts. Findings presented in this study suggested that the polysaccharides extracted from *P. eryngii* mycelium from SMS might be suitable for functional foods and natural antitumor drugs development [16]. Ma et al. [88] used the ultrafiltration technology for purification of polysaccharides and two fractions of polysaccharides from mushroom by-products (CPPS-1 and CPPS-2) with different molecular weight ranges were obtained. After the ultrafiltration membranes used for the preliminary extraction of CPPS, two fractions named CPPS-1 (≥100 kDa) and CPPS-2 (≥10 kDa) were obtained with different molecular weight ranges. The results showed that CPPS-1 had the highest antioxidant activity (65.60 ± 0.87% DPPH scavenging activity, 53.48 ± 5.57% ABTS radical scavenging activity and 87.03 ± 4.21% O_2_ radical scavenging activity), as well as higher cytotoxicity against HepG2 cells compared to other fractions [88].

Glycoproteins are characterized by polysaccharide-peptide or polysaccharide-protein complexes with the protein/polypeptide chains covalently and specifically bound with carbohydrate side-chains. Generally, glycoproteins have different structures and bioactivity mechanisms compared with those of polysaccharides [92]. Some of the common extraction methods to obtain glycoproteins from mushroom by-products are also presented in Table 1.

Lectins constitute a class of proteins with at least one noncatalytic domain that binds specifically and reversibly to different types of glycoproteins, monosaccharides or oligosaccharides [43]. Lectin was isolated from *L. sulphureus* and exhibited a potent exploitable relative agglutinating activity, and can, thus, be developed into a health care product or drug [43]. Further studies must focus on its in vivo bioactivity and relationships between bioactivity and structure.

The studies on extraction of glycoproteins from mushroom by-products are scarce and more research is required to evaluate the possibility of application of these bioactive compounds, especially in the medicine field. The anti-tumor and immunostimulatory activities of water-soluble polysaccharide–protein complexes have been demonstrated in mice models [99]. These compounds can be developed individually as potent biological response modifiers for cancer treatment and also as standard anti-cancer drugs. Further studies on the mechanism by which this complex induces these effects and additional clinical usefulness in therapies of cancer are necessary.

### 3.2. Polyphenols and Related Compounds

Mushroom consumption has increased due to a series of well-known nutritional and therapeutic effects on human health, resulting also from their contents in polyphenols and related compounds [106]. Several extraction protocols, separation and chromatographic methods have been developed to optimize the extraction and identification of phenolic compounds from mushroom waste and by products. The extraction method, solvent type, temperature, extraction time, particle size of the material to be extracted and solid–liquid ratio have impacted the extraction performance. Table 2 shows a summary of selected mushroom by-products from different mushrooms obtained by solid state fermentation as well as submerged liquid fermentation as sources of phenolic compounds.

From these comparative studies, it cannot be concluded that there is an ideal solvent or method to obtain polyphenols from mushroom by-product, as it depends on the combination of the different chosen parameters. Standard chromatographic techniques are the most frequent form of analysis used in the determination of phenolic acids and their related compounds [116]. In general, those by-products continue to be rich in bioactive components, especially in phenolic compounds, and with statistical and mathematical techniques, optimizing the extraction processes could provide for an environmentally friendly and cost-effective condition for producing natural bioactive compounds from these mushroom by-products. Zhu and Xu [109] reported the dynamic production of polyphenols from *Inonotus obliquus*, grown in submerged culture using different lignocellulose materials. Extracts obtained from the fermentation broth and mycelia were found to contain gallic and ferulic acids, epigallocatechin gallate, phelligridin G, davallialactone and inoscavin B. The higher amounts of polyphenols lead to stronger antioxidant capacity displayed by both extracts. Under different culture conditions especially in medium supplemented with H_2_O_2_, several polyphenols were also obtained in an extract prepared from the fermentation broth of *I. obliquus* [111]. Among them are kaempferol (10.9 ± 0.9 mg/g), quercetin (6.7 ± 0.1 mg/g), isorhamnetin (7.8 ± 0.5 mg/g), luteolin (5.8 ± 0.3 mg/g), naringenin (3.4 ± 0.1 mg/g) and apigenin (2.5 ± 0.4 mg/g). The extract also presented strong antioxidant capacity by scavenging both radicals (DPPH and hypoxanthine/xanthine oxidase). Among the phenolic acids found in the mycelial extract prepared from *Suillus bellinii*, by submerged liquid fermentation, p-hydroxybenzoic acid (213 µg/g extract) and cinnamic acid (130 µg/g) extract were the most abundant compounds [112]. 

### 3.3. Steroids and Terpenoids

Among the bioactive compounds found in mushrooms, there is an increasing interest on ergosterol, because of its well-known anti-inflammatory, anti-tyrosinase and anti-cancer [117] activities, together with their use in new drug formulations with antibiotics [118]. Consumption of food formulation with cholesterol reducing properties is an effective strategy to reduce the risks of cardiovascular diseases, with the most widely used bio-based ingredients in commercial products being plant phytosterols [119]. Ergosterol as such because of its similarity to phytosterols, can be utilized to circumvent the severity of high cholesterol [120]. Shimizu et al. [121] identified agarol, an ergosterol derivative obtained from *Agaricus blazei* as the compound was found to induce caspase-independent apoptosis in human cancer cells, making it a promising ingredient for novel anti-cancer formulations. Table 3, Part A shows some common triterpene and related compounds obtained from mushroom by-products.

Heleno et al. [125] studied *A. bisporus* discarded by-products obtained from a local mushroom production company, Mogaricus Cogumelos-Sociedade Unipessoal Lda. Using microwave-assisted extraction (MAE) at the following extraction conditions, 19.4 ± 2.9 min, 132.8 ± 12.4 °C and 1.6 ± 0.5 g/L, an ergosterol yield of 556.1 ± 26.2 mg per 100 g mushroom dry weight by product was obtained. Aqueous methanolic extracts obtained from mycelium of *L. rhinocerotis* produced by solid-substrate was also found to contain several lanosteroid type triterpene and ergosterol [128]. The extract obtained also showed strong antioxidant capacity based on their radical scavenging abilities, reducing properties, metal chelating activities and inhibitory effects on lipid peroxidation. All extracts exerted low cytotoxicity (IC_50_, 200 µg/mL, 72 h) against selected mammalian cell lines. Mycelia culture was obtained from *P. ostreatus* and the culture broth exposed to 2 h of UV-B irradiation. The present ergosterol was converted into vitamin D_2_ content up to 208.65 ± 6.08 µg/g dry weight [134]. Some of these low and high molecular-weights triterpenes and steroids have been reported to be the most important contributors to some health promoting benefits associated with mushroom consumption. Hence these by-products can be viable sustainable alternatives to obtain these above-mentioned metabolites.

### 3.4. Enzymes

Spent Mushroom Substrate is the unutilized substrate and mushroom mycelium left after harvesting of mushrooms [7]. Considering that the substrates for solid substrate fermentation are usually lignocellulosic, protein or starchy residues, most enzymes produced by mushrooms in solid substrate fermentation include cellulases, xylanases, laccases, proteases and amylases [4,20]. The production of lipases, pectinases and phytases can also be significantly explored. These enzymes are usually produced during the early stages of mycelial growth; however, they can also be recovered from the substrate used for the production of fruiting bodies [20].

Industrial by-products of white-rot fungi cultivation, in particular *L. edodes*, *Hericium erinaceus*, *Stropharia rugosoannulata*, *Fomes fomentarius* and *Grifola frondosa*, were screened in terms of composition and selected enzyme activities for potential biorecycling to produce economic profitable enzyme preparations. Spent mushroom substrate of *L. edodes* cultivation was proven as a natural rich resource in enzymes for conversion of lignocellulosic biomass [135].

Mayolo-Deloisa et al. [131] evaluated the potential use of aqueous two-phase systems (ATPS) to recover laccase from the residual compost of *A. bisporus*. The results demonstrated the potential application of ATPS for the valorization of residual material (overall yield of 95%) and the potential establishment of a downstream process to obtain value added products with commercial application [131]. It is believed that the type of enzymes produced by mushroom during cultivation is directly affected by the composition of the growing substrates and by the mushroom species [7,129]. The important parameters to consider for the optimization of the extraction methods on enzyme recovery are pH, temperature, extraction medium, incubation time, inoculum density and nitrogen source [7]. Table 3, Part B includes the extraction methods of enzymes from mushroom by-products.

The recovery of lignocellulose-degrading enzymes from SMS was assessed and maximum recoveries of active xylanase activity were detected in extracts from SMS, which had been physically treated by blending the compost in distilled water [129]. Ko et al. [132] determined the production of amylase, cellulase, glucosidase, laccase and xylanase from the SMS used in the production of four edible mushroom species (*P. ostreatus*, *L. edodes*, *F. velutipes* and *H. erinaceum*), in order to evaluate the potential of using enzymes from SMS as industrial enzymes. The highest xylanase activity was found in the SMS of *F. velutipes* and the enzymes analyzed could be recovered in comparable amounts using different extraction media, whose composition is important for enzyme yields and production costs [132]. 

There is a lack of studies concerning the extraction of enzymes from fermentation broth. Liu et al. [133] described the production, purification and characterization of a novel dual functional fibrinolytic enzyme from the fermentation broth of *Cordyceps militaris*. The enzyme had dual functions, it can act as a fibrinolytic enzyme as well as an anticoagulant and prevent intravascular thrombosis, which is the root cause of cardiovascular diseases [133]. Based on this study, the enzyme had great potential in prevention and treatment of thrombosis.

Spent mushroom substrate is the by-product from mushroom production most reported in the literature for enzymes extraction. It is interesting to verify that, even using a solvent like water, it is possible to recover enzymes with good activity. This fact is of crucial importance when thinking about an industrial application and environmental concerns. The extraction from submerged culture supernatant (fermentation broth) is easier than from SMS, because there is only a need of centrifugation and the supernatant can be used as crude enzyme.

## 4. Potential Application of Mushroom By-Products

### 4.1. Food Uses

Mushroom by-products have a vast potential for food applications, but in this field there is a lack of scientific studies. Mycelium from solid substrate fermentation mushroom production has the potential to be integrated in food products adding functionality, nutritional characteristics and eventually improving sensorial properties. Polysaccharides from edible mushrooms are exceptional target molecules for potential and existing applications in pharmaceuticals, as well as nutraceuticals and functional foods. Their multiple therapeutic properties have been extensively studied, and it seems there is a great potential for exploiting these biopolymers in formulating food to fortify human health [12]. However, the industrial manufacturer of biopolymer-based nutraceuticals and functional foods has several prerequisites that need to be addressed, such as the economically viable production of polysaccharides with stable and standardized quality, composition, purity and homogeneity, the understanding of molecular interactions of bioactive polysaccharides with other food components and the impact of food processing upon their functionality. In addition, more clinical studies on the efficacy of such food products will be required to be able to establish a health claim. Recently, Bilbao-Sainz et al. [136] recovered chitin from mushroom stipe offcuts and converted into chitosan to produce edible coatings. They used a layer-by-layer (LbL) electrostatic deposition of the polycation chitosan and the polyanion alginate to coat fruit bars enriched with ascorbic acid. The bars containing alginate-chitosan LbL coatings showed increased ascorbic acid content, antioxidant capacity, firmness and fungal growth prevention during storage [136]. In addition, LbL assembly is a cheap and simple method that can improve the quality and safety of fruit bars [136]. An American food technology company, Atlast food (https://www.atlastfood.co/), uses solid state fermentation to produce mycelium for meatless alternative products. The mycelium grows up and out of the agricultural waste. The mixture is placed in growth chambers that mimic the conditions of the soil. Mycelium obtained mimic muscle tissue fibers found in whole cuts of meat. This is an interesting approach economically viable, which meets ecological concerns and consumers demand for meat free products. The integration of mushroom by-products in food matrices may confer additional nutritional, sensorial and quality characteristics important for the development of food products. More research is necessary in order to evaluate the economical and processing feasibility concerning mushroom by-products use in food products.

### 4.2. Animal Feed

The occurrence of multiple resistances towards several traditional antimicrobials in animal feed formulations raised concerns. Research is being promoted and performed on natural alternatives. Some research has been conducted in order to use mushroom by-products in animal feed formulation. The nutritive value of SMS from *A. bisporus* mushroom production as ruminant feed was determined. An experiment was conducted, in which four wheat straw-based diets were tested in a cross-over design using eight sheep. Inclusion of a diet containing 30% (*w*/*w*) SMS presented significant lower digestibility. Nitrogen balance was also significantly different between the treatments [137]. Application of SMS from *A. bisporus* for animal feed is restricted according to European regulation [138], because contains animal manure in its composition. Another study was carried out to investigate the effects of fermented king oyster mushroom (*P. eryngii*) by-products diet on pork meat quality characteristics, during refrigerated storage. A mixture of 40% (*w*/*w*) king oyster mushroom by-products, 28% (*w*/*w*) soybean meal and 20% (*w*/*w*) corn were fermented for 10 days, and the basal diet was then replaced by the fermented diet mixture of up to 20, 50 and 80% (*w*/*w*), respectively. The results indicated that the fermented king oyster mushroom by-products diet in swine diet influenced the quality of the meat, it decreased Warner–Bratzler shear force and cooking loss but increased crude protein content, water holding capacity and lightness of muscles in fattening pigs [139]. According to the results, fermented king oyster mushroom by-products may be an economically valuable ingredient. Another study was conducted to investigate the effects of fermented king oyster mushroom by-products diet on the growth performance, blood characteristics and carcass traits of fattening pigs, and also on economic benefit through decreased feed cost. The fermented diet mainly contained 40% (*w*/*w*) king oyster mushroom (*P. eryngii*) by-products, 20% (*w*/*w*) corn, 28% (*w*/*w*) soybean meal, 0.1% (*w*/*w*) supplemental probiotics and 0.08% (*w*/*w*) cellulase. Although the use of fermented mushroom by-products decreased the growth performance, it improved carcass grade and concentration of crude protein, and decreased the feed cost of fattening pigs. Therefore, it was expected that the increase in the percentage use of fermented mushroom by-products would reduce the cost of animal production [140]. The effect of SMS meal on the growth performance and meat characteristics of geese was determined. The results suggested that supplementation of the diet with 5% (*w*/*w*) SMS had no adverse effects on the growth of grower geese. Spent mushroom substrate meal at 5% (*w*/*w*) in the diet favorably affected sensory attributes, namely meat flavor and acceptability [141]. The use of SMS as a replacement to wheat bran on broiler performance was also investigated. The proximate content of SMS compared with wheat bran revealed that wheat bran has appreciable crude protein value than SMS, 17.10 g and 7.88 g per 100 g, respectively. In terms of fiber, SMS was richer than wheat bran with values of 29.57 and 11.25 g per 100 g, respectively. According to the results, further studies must be required to make SMS more profitable in raising broilers, because control diet was cheaper than SMS diet [142].

It has been established that dietary fiber influences the passage rate of digest, it decreases the transition time, the ion exchange and absorption characteristics, leading to the retard of digestion and absorption of nutrient [142]. A feeding trial was developed to investigate the possible effects of supplementation of Nile tilapia diet with *C. militaris* SMS, alone or combined with *Lactobacillus plantarum*, on immune parameters and growth performance. Results on growth performance indicated that fish fed supplemented diets showed a statistically significant increase in the specific growth rate, weight gain and final weight compared to the control group [143]. The highest specific growth rate and weight gain values were observed in fish fed both dietary SMS and *L. plantarum* suggesting that the combination of dietary SMS and *L. plantarum* could be considered as potential feed-additives for aquaculture farmed fish [143].

In animal feed, the main by-products used for feeding trials are from solid substrate fermentation. According to the available studies, the advantages associated with mushroom by-products supplementation for animal feed are related to the animal quality, economic viability and ecological concerns.

### 4.3. Fertilizer and Partial Substrate for Mushroom Growth

Mushroom production industry has a challenge on the management of SMS, and research is being developed towards environmentally and economically sustainable solutions for this organic residue. Due to its physical properties and nutrient content, SMS has great potential to be employed in agricultural or horticultural sectors. However, SMS is often regarded as not being stable and/or mature, which interfere its wide use for crop production. A study demonstrated the stabilization of SMS and its subsequent use as organic fertilizer and partial peat replacement in horticulture [144]. The stabilization was performed in a laboratory-scale composting system, with controlled temperature and aeration. Maturity and quality of the stabilized SMS were assessed in a horticultural growing trial. When used as the sole fertilizer source, SMS was able to support *Lolium multiflorum* Lam. (Italian ryegrass) growth and significantly improved grass yield. The results indicated that the method developed was efficient in generating a stable and mature product, which has a potential to be applied in horticulture [144].

Cultivation waste from mushroom production is considered to have several advantages, namely low collection cost if used by the same production facility, and it contains many inorganic nutrients [145]. Mushroom culture waste may contain plant nutrients (P, K, Mg and Si) and ash from such biomass waste can be used as fertilizer. Kim et al. [145] suggested a new concept for a more affordable method of using this waste, namely, cascade use of enokidake mushroom culture waste as fuel, followed by the use of the ashes as fertilizer. Spent mushroom substrate can be used as seedling cultivation media or can be added into the media at certain rates. However, salt content could be a restrictive factor in the determination of the amount of use [146].

Owaid et al. [147] mentioned the role of some effective date palm wastes, containing fibers of date palm, mixed with white sawdust and wheat straw in three formulas, in improving SMS properties. The experiment was carried out to study the suitability of SMS to the production of edible plants or edible mushrooms. Spent mushroom substrate can be integrated using new formulations and methodologies with the added advantages of lowering production costs and decreasing the environmental impact of its ever-growing accumulation. Spent mushroom substrate may be used in bio-organic applications, as a type of bio-fertilizer, due to the formulation of carbon sources and rock phosphate used [147].

Some studies reported SMS utilization for mushroom production. Mixtures of coconut fiber pith and SMS were studied as casing material in mushroom cultivation. The results indicated that this alternative could be considered to partially replace the organic substrates normally used for mushroom cultivation, with the advantages of decreasing cost and reducing the environmental impact of waste disposal [148]. A study demonstrated that SMS can be used as a potential substrate for mushroom production of *Pleurotus* spp. and supplementation of SMS with sawdust and wheat bran can produce even higher yields. Spent mushroom substrate alone was not able to produce good yield of mushroom, because depletion of nutrients occurred in the substrate due to subsequent utilization of nutrients by mushroom mycelium. The sawdust and wheat bran added SMS produced higher yields compared to SMS alone [149]. However, the use of SMS for mushroom production is dependent on mushroom specie and nutrients requirement for growth.

From all the by-products resulting from mushroom production, SMS is the only one on which some research was performed concerning fertilizer application. This may be due to the major environmental concern associated to disposal of this material. Some research has been developed showing the potential of SMS application as fertilizer and promising results indicate that a combination of materials is required to obtain higher production yields.

### 4.4. Bioremediation and Biological Treatments

Some studies report the use of SMS for bioremediation purposes by degrading pollutants in the environment through extracellular enzymes. Spent mushroom substrate was tested for acetaminophen and sulfonamides removal, which are emerging contaminants. Among the SMSs obtained from production of nine mushrooms, tested in batch experiments, the SMS of *P. eryngii* exhibited the highest removal rate for acetaminophen and sulfonamides. The results of this study provided a feasible solution for acetaminophen and sulfonamide removal from wastewater using the SMS of *P. eryngii* [150].

Pentachlorophenol (PCP) has been used as a wood preservative. Although it has been banned worldwide, residues of PCP are still commonly found. The SMS of oyster mushroom *Pleurotus pulmonarius* was able to degrade PCP. The maximum removal capacity was 15.5 ± 1.0 mg/g SMS. One isolated bacterium from SMS with unknown identity could tolerate PCP at 100 mg/L and was inoculated to a medium at a final concentration of 7.5 × 10^7^ cfu/mL. Although PCP-degradative bacterium was isolated from SMS, biodegradation was predominantly realized by immobilized ligninolytic enzymes secreted by mushroom to the SMS [151].

Lau et al. [152] obtained complete removal, by degradation, of individual naphthalene, phenanthrene, benzo[*a*]pyrene and benzo perylene by 5% SMS of *P. pulmonarius* in two days at 80 °C. The results demonstrated the potential of employing SMS in ex situ bioremediation [152]. Another recent study reported SMS, ochre, steel slag and limestone as effective agents in removing heavy metals and increasing the pH of the acid mine drainage. The mixed substrates SM1 (i.e., 10% (*w*/*w*) SMS mixed with 20% (*w*/*w*) ochre, 30% (*w*/*w*) steel slag and 40% (*w*/*w*) limestone) and SM2 (i.e., 20% (*w*/*w*) SMS mixed with 30% (*w*/*w*) ochre, 40% (*w*/*w*) steel slag and 10% (*w*/*w*) limestone) were effective in increasing the pH 3.5 to 8.09, and removing heavy metals with more than 90% efficiency [153].

Another field that has been explored in bioremediation is the dye removal through the enzymatic activity of SMS from solid substrate fermentation and fermentation broth submerged liquid fermentation, respectively. Until now, the treatment methods of dye wastewater included mainly physical, chemical and biological methods. The biological treatment method had been widely concerned and studied because of its low processing cost and environment friendliness [154]. As a result of the abundant surface hydroxyl, carbonyl, carboxyl, amide, phosphate groups in SMS, it could be considered as an appreciable low-cost adsorbent for wastewater treatment [154]. In textile dyeing considerable amounts of dyestuff, e.g., up to 30% of reactive dyes, are lost and discharged with the effluents [155]. Therefore, elimination of dyes from textile dyeing effluents currently represents a major ecological concern. Mushrooms are known to degrade textile dyes due to the unspecific nature of their lignin degrading enzymatic system [156,157,158]. Peroxidases and laccases are the enzymes responsible for this action. The fact that these enzymes are excreted by the mushroom makes these organisms especially interesting for bioremediation [155]. Lim et al. [4] conducted a study in order to perform efficient extraction of lignocellulolytic enzymes amylase (EC 3.2.1.1), cellulase (EC 3.2.1.4), laccase (EC 1.10.3.2) and xylanase (EC 3.2.1.8) from SMS of *Pleurotus ostreatus*, *P. eryngii* and *Pleurotus cornucopiae*. Enzymatic activities varied according to the SMS released from different mushroom farms. The synthetic dyes remazol brilliant blue R and Congo red were decolorized completely by the SMS extract of *P. eryngii* within 120 min, and the decolorization ability of the extract was comparable to that of 0.3 U of commercial laccase. In addition, laccase activity of the SMS extract from *P. eryngii* was compared to that of commercial enzymes or its industrial application in decolorization [4]. A laccase (EC 1.10.3.2) was isolated from the liquid culture filtrate of *L. edodes*. The enzyme was purified through a homogeneous preparation using hydrophobic, anion-exchange and size-exclusion chromatography. The enzyme has oxidized 2,2′-azino-bis(3-ethylbenzothiazoline-6-sulfonic acid) diammonium salt, *p*-phenylendiamine, pyrogallol, guaiacol, 2,6-dimethoxyphenol, catechol and ferulic acid, but not veratryl alcohol, tyrosine or β-(3,4-dihydroxyphenyl) alanine. The N-terminal amino acid sequence of laccase showed close homology to the *N*-terminal sequences determined for laccases from *Phlebia radiata*, *Trametes villosa* and *Trametes versicolor*, but only low similarity was observed to a previously reported laccase from *L. edodes*. Laccase was effective in the decolorization of chemically different dyes—remazole brilliant blue R, bromophenol blue, methyl red and naphtol blue black—without any mediators, but the decolorization of two dyes—red poly(vinylamine)sulfonate-anthrapyridone dye and reactive orange 16—required some redox mediators [159]. Recently, a novel extracellular laccase was purified from fermentation broth of the white rot fungus *Trametes* sp. F1635 by a three-step protocol including two consecutive ion-exchange chromatography steps on DEAE-Sepharose and SP-Sepharose, and a final gel-filtration on Superdex 75. The purified laccase demonstrated high oxidation activity. Purified laccase showed effective decolorization activity towards eriochrome black T, remazol brilliant blue R, malachite green and eriochrome black T (over 60%). Violuric acid and acetosyringone were the optimal mediators for the laccase in dye decolorization [160]. The treatment of wastewater always demands eco-friendly and cost-efficient adsorbents. With this purpose, SMS was modified by a cationic surfactant (cetyltrimethylammonium bromide, CTAB) to eliminate toxic dyes [39]. The characterization of the adsorbents confirmed that CTAB was successfully embedded into the SMS structure. The SMS from *P. ostreatus*, modified by CTAB (SMWC), exhibited an excellent adsorption capacity for the Direct red 5B, Direct blue 71 and Reactive black dyes. Batch experiments indicated that the dye adsorption of SMWC depended mainly on pH, dye concentration, temperature and ionic strength. Therefore, SMWC could successfully remove over 90% of dyes from various water samples. This was considered a feasible waste resource utility, since it meets both the ecological and the economic requirements for auspicious industrial applications [39].

Spent mushroom substrate of *G. lucidum* (SMS-GL) was firstly used as a bio-adsorbent to adsorb three typical dyes, malachite green, safranine T and methylene blue, and the adsorption thermodynamics and dynamics were also studied. The by-product SMS-GL was rich in hydroxyl groups and carbonyl groups, presenting the potential to be an efficient bio-adsorbent for the three dyes removal from water and wastewater, and the treatment model should be eco-friendly. In addition, SMS-GL could adsorb the dyes rapidly and achieve an equilibrium in a short time [154]. In turn, Nakajima et al. [3] studied the active enzymes recovery from SMS of *Hypsizygus marmoreus*, *F. velutipes*, *P. eryngii*, *L. edodes, P. ostreatus* and *Pleurotus* sp. Different types of enzymes (exocellulase, endocellulase, total cellulase, β-glucosidase, dextranase, amylase and laccase) were recovered from the various SMS, demonstrating the diversity of enzymatic activity in these compounds is dependent on the mushroom species. It was also observed that the extract obtained from *Pleurotus* sp., had a different enzymatic profile, with a higher decolorizing capacity than other fungus tested. The results indicated that a specific type of Basidiomycete SMS should be selected, depending on the desired enzymatic activity, since each one presents a distinct enzymatic profile [3].

Juárez et al. [161] investigated the use of SMS on the degradation of chlorothalonil in agricultural effluents. SMS extract was capable of reducing the initial concentration of chlorothalonil (2 mg/L) by 100% after 45 min of reaction. The study revealed that storage time, cooling and freezing had a negative effect on the stability of enzymatic activity [161]. More research is needed to optimize the use of SMS, otherwise, the applicability of this by-product as a pesticide degrader may be compromised.

The use of agricultural wastes as biosorbents is achieving importance in bioremediation of heavy metal-polluted water and soils, due to their efficacy and low cost. The study of Frutos et al. [162] assessed the Cd, Pb and Cu adsorption capacity of the raw materials employed in the production of substrates for mushroom production (*A. bisporus* and *P. ostreatus*) and the SMS, based on the functional groups of their organic carbon. The results were valuable to develop new biosorbents based on agricultural wastes and demonstrated the high potential of SMS to adsorb heavy metals from polluted environments [162].

Summarizing, dyes can be removed, degraded and detoxified by enzymatic biological process and also physical adsorption by using SMS [163]. SMS in bioremediation of dye is definitely more time saving and cost effective [7]. The major limitation with SMS is its disposal after adsorption. There is scarcity of reports on the fate of SMS after adsorption. As the SMS is loaded with high amount of pollutants and heavy metals, it needs to be treated and disposed in a proper way in order to prevent the spread of its toxicity in the environment [164]. Additionally, fermentation broth from mushroom production appears to be an interesting by-product with decolorization activity, resulting from the available enzymes excreted from mushrooms.

### 4.5. Energy Production

Spent mushroom substrate can be burnt to produce energy [165]. Burning is a type of combustion with the formation of flame and lower amount of heat energy. Spent mushroom substrate often contains high ash content, which makes the process less efficient and leads to new waste problems [6]. For instance, *A. bisporus* is grown on compost which contains around 50% of ash on a dry matter basis [166]. In comparison, for *Pleurotus* spp. which is cultivated on sterilized sawdust based substrate the ash content is lower, around 6% of ash on a dry matter basis [167]. The compositional difference in these two types of spent mushroom substrate will affect its applications.

Alternatively, SMS can be subjected to combustion, which is the most effective process, because it is self-sustaining and generated temperatures can be used for the production of saleable heat and/or power [6]. Lignocellulosic material is regarded as a promising energy source because it is renewable and consists of abundant carbohydrates [168]. The SMS is useful in a fermentative process, such as that leading to the production of bioethanol. Some studies are reported in this context with different types of mushroom substrates and mushroom species, leading to different ethanol volumetric productivities. Nowadays, the cost of production of bioethanol is relatively high. The use of SMS could be a strategy for improving the yield and reducing the costs of the process [169]. Normal wood and SMS were examined towards their physico-chemical characteristics by Lee et al. [170] in order to investigate the opportunity of using SMS as an alternative biomass resource for energy production. After 24 h fermentation, 12 g/L ethanol was produced with SMS, while normal wood generated 8 g/L ethanol. Spent mushroom substrate showed potential as an economical alternative energy source, because of its easy conversion to fermentable sugars through various hydrolysis methods and low energy consumption during size reduction. Additionally, SMS was ready for hydrolysis with only mild or no pretreatment because of an efficient pretreatment with extracellular enzymes of *L. edodes* during cultivation, leading to a lower lignin content and crystallinity. These results indicated that SMS was an economically suitable lignocellulosic material for the production of fermentable sugars related to bioethanol production [170]. As a further study, the authors considered that heterogeneity of SMS was regulated and problems about collection as well as conveyance of biomass must be resolved for practical application in a real industrial case.

In another study, SMS was hydrolyzed using concentrated sulfuric acid, and its performance in fermentative hydrogen production from the hydrolysate was investigated. Biohydrogen production was successful by using the hydrolysate of lignocellulosic materials from mushroom farm waste, which removed the sulfate ions from the reduced sugars solution with an anion exchange resin [171].

Spent mushroom substrate can be a suitable feedstock for biogas production, but high degradability of SMS can exert some adverse influence on anaerobic digestion. Recently, the methane yield and microbial community resulting from co-digestion of SMS and dairy manure at different mixing ratios was evaluated. Results indicated a synergistic effect of co-digestion of SMS with dairy manure, reflected in a higher methane yield [172]. Butanol production main challenges include high cost of traditional feedstock and fermentation inhibitory compounds which affects bacterial growth and consequently butanol efficiency [173,174]. Spent mushroom substrate was evaluated as a potential lignocellulosic substrate for butanol production using in situ removal by biodiesel. An improved organic solvent method was first developed for SMS pretreatment under non-corrosive and mild conditions by replacing ethanol with water alone in the washing steps. The batch fermentation results revealed that the addition of biodiesel can successfully enhance butanol production from SMS enzymatic hydrolysate, and the fed-batch operation, with in situ butanol removal, by biodiesel greatly enhance butanol productivity. The combination of improved economic pretreatment process and successful biodiesel extraction system make SMS, the low-cost feedstock, present a great potential of application in the scale-up of butanol fermentation [175].

Recently, a very interesting review was published, proposing to integrate SMS from mushroom production into a vicious circle of resource reutilization for energy generation, particularly as a substrate for biogas generation [176]. The authors noted that there is a real necessity for the optimization of important factors that influence the yield of biogas such as, type of biomass, amount of volatile solids, optimum proportion of substrates and inoculums in either mono or co-digestion and mushroom species employed.

### 4.6. Cosmetics and Cosmeceuticals

Cosmeceuticals represent the newest trend in the skin care industry, and they are mainly products in the form of cream and lotions with biologically active compounds, that influence the biological function of the skin by supplying the needed nutrients for healthy skin [177]. Several bioactive metabolites from the large molecular weight polysaccharide and glycoproteins to other metabolites such as phenolic compounds, terpenoids, steroids and several lipid components have been associated with skin health.

Several extracts obtained from mushroom by-products, as well as their secondary metabolites, have been reported to display important biological functions such as antioxidant, anti-inflammatory, antitumor, antimicrobial, anti-ageing and photoprotective effects [24,178,179]. As a result of the negative effect associated with the synthetic ingredients, the cosmetic industry is constantly, in search for alternative natural active biomolecules with the ability to treat these disorders, such as the ones obtained from mushroom by-products. From a sustainable point of view, residues produced during mushrooms production processes remain an important source of bioactive metabolites that can be utilized as cosmeceutical ingredients. 

Mycelia extract prepared from *Lentinus polychrous* at 100 μg/mL, was found to maintain cell viability of RAW 264.7 macrophage cells while reducing the expressions of different inflammatory mediators [180]. Mycelia extract were obtained from *A. blazei* and was found to inhibit tyrosinase activity and melanin content in B16F10 melanoma cells up to 49 and 45% at 10 and 0.75 mg/mL, respectively. The expression of melanogenesis-related proteins microphthalmia-associated transcription factor (MITF), tyrosinase, tyrosinase related protein-1 (TRP-1) and tyrosinase related protein-2 (TRP-2) were significantly suppressed, with results comparable to kojic acid and arbutin used as positive controls. This makes *A. blazei* mycelial extract a potential ingredient to suppress the severity of hyperpigmentation [181]. Similarly, mycelia extract from *G. lucidum*, *A. camphorata*, *A. blazei* and *C. militaris* were submitted to anti-tyrosinase activity, with *G. lucidum* showing up to 80% tyrosinase inhibition at 0.32 mg/mL [182]. *A. blazei* residues rich in fatty acids, mannitol and ergosterol were utilized as a cosmeceutical ingredient. Ethanolic extract prepared from its residue was found to present anti-tyrosinase activity while also maintaining cell viability effects in HaCaT cell line (keratinocyte) in a concentration dependent manner [183]. Dr. Andrew Weil for Origins^TM^ mega-mushroom (www.origins.com/dr-weil-mega-mushroom) was among the first premium western brands to exploit mushroom in skin care. In its catalogue is an anti-ageing cream formulated with *Hypsizygus ulmarius* mycelium, in the presence of other bioactive extracts. Some of these findings have shown that by-products obtained from mushroom can be valuable sources of bioactive extracts or individual metabolites to be incorporated in topical formulations to maintain skin health. 

### 4.7. Bio-Based Materials

Mycelium seems to be an interesting by-product for development of bio-based materials, mainly because of its composition and structure. Mycelium has a filamentous network structure with mechanics largely controlled by filament elasticity and branching, and network density [184]. Mycelium composites use biological growth rather than expensive energy processes to convert low-cost organic wastes into economically viable and environmentally friendly materials [185]. Since many developed countries are progressively adopting the use of sustainable materials as a strategy to reduce environmental pollution, mycelium-based materials highly support this strategy. The developed mycelium-materials require minimum energy for production (self-growing), and their characteristics can be tuned by modifying their nutrient substrates [186]. A range of potential applications have been proposed including acoustic dampers, super absorbents, paper, textiles, structural and electronic parts [185].

Mechanical properties of the mycelium are impacted by genetic modification and environmental growth conditions affecting the density of the mycelium [187]. Wild type mycelium was similar leather, while the genetically modified was more similar to thermoplastics (e.g., polyethylene, polypropylene, polyvinyl chloride) [187]. This was mainly caused by increased density and not by increased strength of the material. This work showed that fungal materials can be further modified by treating mycelium chemically or physically in order to obtain the characteristics of interest. A fungal mycelium-based biofoam was obtained by three different mixing protocols with various substrate materials, including wood pulp, millet grain, wheat bran, a natural fiber and calcium sulfate, and two packing conditions. The results demonstrated a great potential for application as an alternative insulation material for building and infrastructure construction, particularly in cold regions as light-weight backfill material for geoengineering applications [188].

Islam et al. [184] presented morphological and mechanical characterization of a novel biomaterial derived from fungal mycelium. The experimental results revealed the most significant characteristics of mycelium under tension and compression. Under tension, the material response is linear elastic at low strain, and then the material undergoes strain hardening before rupture [184]. Visual appearance, density, mechanical properties and water-absorbing behavior were assessed for a range of mycelium-based composites that were obtained by varying the type of fungus, substrate and pressing conditions. *P. ostreatus* and *T. versicolor* colonized the substrate, but also formed a fungal skin (air-exposed mycelium) at the substrate–air interface [189]. The fungal species impacted colonization level and the thickness of the fungal skin. Colonization level and skin thickness, as well as the type of substrate, determined the stiffness and water resistance of the materials. Moreover, it was shown that heat pressing improves their homogeneity, strength and stiffness, shifting their performance from foam-like to cork- and wood-like. Together, these results demonstrated that, by changing the fabrication process, differences in performance of mycelium materials can be achieved [189].

For the last years, research has been conducted on fungi based mushroom packaging material as an alternative to conventional plastic and cost competitive to any other standard foams. Polystyrene foam whose main component is derived from petroleum or natural gas, is a prominent packaging material, which is neither biodegradable nor compostable [190]. Incorporating mycelium based materials for packaging could help in reducing polystyrene foam consumption, and would eventually lead the way for eco-friendly packaging, thus, enhancing sustainability without any compromise on cost or performance [190].

Spent mushroom substrate was studied as a new additive to produce bricks with better insulation. The aim was to determine how SMS changes properties of fired clay bricks, the thermal behavior, and if it is a viable option for recycling SMS. Addition of 17% of SMS leaded to a 26.17% decreasing in thermal conductivity compare to those without SMS, achieving a minimum thermal conductivity of 0.55 W/m K. This implied a reduction of 10% on the equivalent thermal transmittance, which means a better insulation of the buildings and, thus this was considered an important energy saving. Fired clay made with SMS enhanced thermal properties of bricks while water absorption and compressive strength values were according to standards. However, bricks could be non-accepted by market due to such low compressive rupture strength and, therefore, this may limit the addition of amount of SMS [191]. In addition, the authors identified a limitation concerning organic gases, which could make mandatory an exhaust gases treatment installing. For this reason, it was recommended to analyze this issue before producing it at a real scale.

An US-based startup named Ecovative (https://ecovativedesign.com/) produce mycelium-based materials and products. One of the developed products is Myco board to replace wood. This material is compressed with heat and pressure into boards, and no chemical resins are used. The product is considered cheap, with efficient production, which helps everyone to achieve zero waste. The company Mycoworks (https://www.mycoworks.com/) from San Francisco (USA) created a new kind of leather based on mushroom mycelium and agricultural by-products in a carbon-negative process. The material is considered sustainable, versatile and animal-free and it feels and performs like leather. Ecovative also developed a mycelium based biodegradable packaging to replace petroleum based ones [192]. Mycelium based material has been studied for food packaging as a sustainable alternative for polysterene [190].

These biomaterials represent a promising alternative for product design and manufacturing, both in terms of sustainability and circular economy. Recently, some companies are exploring mycelium-based materials revealing great potential in this field. Limited research, inconclusive data and the proposed applications and feasibility suggest that further investigation is warranted, specially exploring mushroom by-products, such as SMS.

## 5. Regulatory Challenges in Europe

The potential valorization of mushroom by-products through the extraction of new functional ingredients application in new foods have regulatory implications. The European Food Safety Authority (EFSA) is responsible for evaluating the scientific evidence supporting health claims. Health claims are only authorized after a scientific assessment of the highest possible standard, registration of nutrition and health claim. A search of all bioactive compounds mentioned above (Section 3) was performed to evaluate the potential of application in food products and positive health related effect. Some of the bioactive compounds that can be extracted from mushroom species, although being supported by some health claims reported in studies, they are not yet approved by EFSA, so they are associated to non-authorized health claims (Table 4). This means that for some bioactives reported for mushroom species, the claimed effect for the food is not a beneficial physiological effect as required by the Regulation [193].

On the other hand, there are also some health claims associated to some of the bioactive compounds commonly found in mushroom by-products that have been approved by EFSA and are registered on nutrition and health claims (Table 4). However, the approval of specific bioactives from a specific source may not be directly applicable to other sources. A search of all bioactive compounds mentioned above (Section 3) was performed to evaluate the bioactives with potential health benefits that could be applied as functional ingredients in food products with an approved health claim (Table 4). The main bioactive compounds found were beta-glucan, chitosan and sterols. In fact, although there are several studies reporting the biological activity of these compounds, scientific evidence is crucial to proceed with these products towards the market and particularly with an approved health claim by EFSA. Scientific proofs are derived from in vitro studies, but mainly in vivo studies and clinical trials. For instance, in the case of beta-glucans, initially the approved health claims mentioned that 3 g of soluble fiber from oats reduced cholesterol by 0.13 mmol/L, whereas more recent analyses indicate a reduction in low density lipoprotein (LDL-C) of ~ 0.27 mmol/L for a similar intake; a fourth claim relates to reduction in post-prandial glycaemia with the consumption of 4 g beta-glucans/30 g available carbohydrate [194]. However, these claims can only be applied if the source is beta-glucan from oats or barley. This may open the opportunity to expand the R&D studies to demonstrate the benefits of mushrooms beta-glucans to eventually include this source in the list of approved health claims in future. In fact, an aqueous mycelial extract of *Lentinula edodes* composed of beta-glucan lentinan, free glucose and N-containing constituents—Lentinex^®^—was evaluated by EFSA. The safety of Lentinex^®^ as a novel food ingredient has been established at specific proposed conditions of use and the proposed levels of intake (intake of 2.5 mL Lentinex^®^ containing 1 mg lentinan (beta-glucan)/mL correspond to 41.7 μg/kg body weight for a 60 kg person). Although, this product was successfully evaluated for safety purposes [195], no health claims associated to beta-glucan lentinan are reported.

In the case of chitosan, the panel considers that in order to obtain the claimed effect “relates to the maintenance of normal blood LDL-cholesterol concentrations”, 3 g of chitosan should be consumed daily just in adults. In this case, they specify that must be O-carboxymethyl Chitosan, but they do not limit the source, so the mushroom derived chitosan may be considered, with the potential advantage of less allergenic issues compared to crustaceous source [196]. Concerning sterols and stanol esters the panel concludes that sterols and stanol esters at a daily intake of 3 g (range 2.6–3.4 g), but in this case, they regulate just for the plant sterols and stanol esters and also for the matrices of application so sterols/stanols they can only be applied in matrices approved by the Regulation (EC) No 376/2010 (yellow fat spreads, dairy products, mayonnaise and salad dressings) lower LDL-cholesterol by 11.3% (95% CI: 10.0–12.5) [197,198].

The regulatory issues are relevant to guarantee the safe use of nutritional and health claims, and may also incentives deeper R&D studies of some of the molecules extracted from mushroom losses or by-products, which assuring absence of toxicity and scientific supported benefits may in future enlarge the list of health claims.

## 6. Conclusions

Mushroom production involves a great variety of by-products with applications in different fields. Bioactive compounds obtained from mushroom by-products is an underexplored topic but with a great potential of application in nutraceutical and food products. There is a demand for environmentally friendly and economic sustainable extraction methods that combined with applicable legislation and the cost of alternatives will turn the up-scaling process and industrial application possible. Bioactive compounds have been extracted and characterized from the different mushroom production by-products—fruiting body (caps and stipes), SMS, mycelium and fermentation broth. Polysaccharides represent the most explored ones, considering all by-products from mushroom production. For instance, in the case of enzymes the majority of the studies are focused on SMS and fermentation broth is not well studied for the extraction and characterization of these compounds. Animal feed, fertilizer, cosmeceutical development, bioremediation and bio-based materials as well as energy production are fields that have been explored in the last years with promising and very interesting results. Bio-based materials constitute an emerging field, which indicates an enormous potential of SMS and mycelium in the development of novel biomaterials with sustainable practices and very interesting characteristics meeting the circular economy concept.

## Figures and Tables

**Table 1:** Extraction methods of polysaccharides and glycoproteins from by-products obtained by solid substrate fermentation and submerged liquid fermentation.

Production Technique	Species	By-Products	Bioactive Compound	Extraction Method	Fractionation and Purification	Bioactivities	Reference
Solid Substrate Fermentation	*Agaricus bisporus*	Dried and powder caps and stipes	Chitin	Alkali treatment (2 M sodium hydroxide, 2 h at 100 °C); Oxalic solution treatment (1% *w*/*v*, 1 h at 100 °C); Alkali treatment (2 M sodium hydroxide, 2 h at 100 °C); Acidic treatment (2% acetic acid, 90 °C for 2 h).	nd	Antimicrobial activity	[84]
		Dried and powder caps and stipes	Beta-glucan	Ultrasound-assisted extraction; precipitation with ethanol 80% (1:2 *v*/*v*, 1 h at room temperature).	nd	nd	[18]
		Dried and powder stipes	Chitosan	Precipitation with 96% ethanol; Treatment with sodium metabisulfite; Treatment with alkali (2% sodium hydroxide, 56 °C for 2 h) NaOH added to the insoluble phase and treatment with hydrogen peroxide (final concentration 3%, stirring at 45 °C for 0.5 h); Treatment with sodium hydroxide (2 h at 104 °C); Treatment with hydrochloric acid.	nd	Good film-forming properties with antimicrobial activity	[85]
		Dried and powder stipes	Chitin-glucan complex	Alkali treatment (1 M sodium hydroxide, 95 °C, reflux 30 min); Acidic treatment (2% acetic acid, 95 °C, reflux 6 h).	nd	nd	[15]
	*Flammulina velutipes*	SMS	Polysaccharide	Hydrochloric acid extraction (0.5 M, 1:10, *w*/*v*), sodium hydroxide (0.5 M, 1:10, *w*/*v*) at 80 °C and snailase solution (4%, 1:4, *w*/*v*) at 37 °C for 6 h, respectively. After centrifugation (3600 r/min, 15 min), the supernatant was mixed with 3 volumes of ethanol (95%, *v*/*v*) at 4 °C overnight.	Sevag method	Antioxidant activity; hypoglycemic and renoprotective effects	[40]
	*F. velutipes*	Dried and powder mycelium from SMS	Polysaccharide	Hot water extraction (1:20 *w*/*v*) for 8 h at 70–80 °C; Concentration in a rotary vapor.	Sevag method; Dialysis; DEAE–Sephadex elution; Sephacryl S-400 column elution.	Antitumor activity	[86]
	*Ganoderma lucidum*	Dried and powder SMS	Polysaccharide	Water extraction (1:35 (*v*/*w*)); Precipitation (20 h).	Sevag method; DEAE cellulose colum; Sephadex G-100 size-exclusion chromatography.	Antioxidant activity	[44]
	*Laetiporus sulphureus*	Stipe	Lectin	Sodium chloride concentration, 0.50; liquid−solid ratio, 20:1 (mL/g); extraction temperature, 35 °C; extraction time, 20 h; and extraction pH, 7.5.	Dialysis; DEAE-cellulose-52; HPGFC (High Performance Gel Filtration Chromatography)	Agglutinating activity	[43]
	*Lentinus edodes*	SMS	Lentinan	Hot water extraction (80 °C for 1 h); Ethanol precipitation (95%); Ethanol washing (75%).	Sevag method; Dialysis (30 °C for 16 h).	Antibacterial activity	[45]
	*L. edodes*	Dried and powder SMS	Polysaccharide	Hot water extraction (1:20 (*w*/*v*), 100 °C, 100 min, repeated 3 times).	Deproteinization–combination of Sevag method and enzymatic hydrolysis (papain); Sephadex G-150 size exclusion chromatography.	Antioxidant activity	[87]
	*Pleurotus eryngii*	SMS	Heteropolysaccharide (mainly composed of xylose, glucose and arabinose)	Alkali treatment (0.5 M NaOH, 90 °C for 300 min); Ethanol precipitation (65%).	Sevag method Dialysis (24 h at room temperature); Ethanol precipitation Sepharose CL-6B column chromatography.	Antioxidant activity	[41]
	*P. eryngii*	Dried and powder mycelium from SMS and fruiting bodies	Polysaccharide	Precipitation with 85% ethanol; Ultrasonic extraction (70 °C, 140 min); Precipitation with ethanol (12 h, 4 °C).	Dialysis; Ultrafiltration (membranes cut off of 100 KDa and 10 KDa).	Antioxidant activity	[88]
	*P. eryngii*	Dried and powder mycelium from SMS and fruiting bodies	Heteropolysaccharide (mainly composed of glucose)	Precipitation with 85% ethanol at room temperature for 12 h; Hot water extraction (70 °C for 140 min).	Sevag method; Dialysis.	Antitumor activity	[16]
Submerged Liquid Fermentation	*Agaricus blazei*	Fermentantion broth and mycelium	Polysaccharide	Hot water extraction (boiling, 1 h); Precipitation with 95% ethanol.	DEAE column; Gel filtration; chromatography HW-65F column.	nd	[89]
	*Antrodia cinnamomea*	Mycelium	Antrodan	Supercritical Fluid Extraction (reflux 3 times, 90 °C for 2 h); Hot alkali treatment (80 °C, 3 times for 2 h); Ethanol precipitation (95%).	Gel Permeation Chromatography (Sephadex G-100 Column)	Anti-inflammatory activity	[90]
	*Coprinus comatus*	Mycelium	Polysaccharide	Microwave for 3 min; Hot water extraction (1:2 *w*/*v*) for 4 h at 70 °C; Concentration in a rotary vapor.	Sevag method; Dialysis in a DEAE cellulose bag; Precipitation with ethanol (48 h at 4 °C).	Antioxidant activity	[91]
	*Grifola frondosa*	Mycelium	Glycoprotein	Extraction with water (4 °C); Precipitation with 80% ammonium sulfate (overnight at 4 °C).	Dialysis; DEAE-Sepharose.	Antitumor activity	[92]
	*Hericium erinaceum*	Mycelium	Polysaccharide (heteroglycan)	Hot water extraction (70 °C for 12 h); Precipitation with ethanol (80%).	Hollow-fiber ultrafiltration; Ion-exchange chromatography (DEAE-Sephadex column).	Antichronic atrophic gastritis activity	[93]
	*H. erinaceum*	Mycelium	12b-hydroxyverruculogen TR-2, fumitremorgin C and methylthiogliotoxin, two hetero-spirocyclic glactam alkaloids, pseurotin A and FD-838 and cerevisterol and herierin IV	Ultrasound assisted extraction with ethyl acetate.	Column chromatography over silica gel Sephadex LH-20; reversed-phase silica gel column chromatography; preparative thin-layer chromatography (PTLC).	Antioxidant and antifungal activities	[94]
	*Inonotus obliquus*	Fermentation broth and mycelium	Polysaccharide	Fermentation broth:Extraction with 95% (*v*/*v*) ethanol (4 °C, overnight); Treatment with Neutrase (3% *w*/*w*, 42 °C for 3 h). Mycelium: Hot water extraction (3 h at 121 °C); Ethanol precipitation.	Sevag method; Dialysis; DEAE-52 chromatography; Sephadex G-200 chromatography.	Antioxidant and immunomodulator properties	[95]
	*I. obliquus*	Fermentation broth and mycelium	Polysaccharide	Fermentation broth: Ethanol precipitation (95%, overnight at 4 °C). Mycelium:Hot water extraction (3 h in an autoclave at 121 °C); Ethanol precipitation.	Fermentation broth: Sevag Method. Mycelium: Dialysis.	Antioxidant activity	[96]
	*Lentinus squarrosulus*	Fermentation broth and dried mycelium powder	Polysaccharide	Ethanol precipitation; Cold water extraction (3 h at room temperature); Hot water extraction (3 h at 100 °C).	nd	Antitumor and antioxidant activities	[97]
	*Morchella conica*	Fermentation broth	Exopolysaccharides	Centrifugation at 3000 rpm for 20 min from the culture; Sevag method;95% ethanol precipitation.	Dialysis; DEAE-Cellulose 52 anion-exchange chromatography column.	Immunomodulatory activity	[98]
	*P. ostreatus*	Mycelium	Proteoglycan	Phosphate-buffered saline treatment and centrifugation (8000 rpm for 15 min); Ethanol precipitation.	Dialysis; Anion exchanger column (DEAE-Sephadex); Cation exchanger column (CM-Sephadex); Gel filtration chromatography (Sephadex G-100)	Antitumor activity	[99]
	*Pleurotus tuber-regium*	Mycelium	Polysaccharide	Hot water extraction (100 °C for 3 h); Ultrasound assisted extraction (2 h).	Dialysis (2 days)	Antitumor activity	[100]
	*Schizophyllum commune*	Fermentation broth	Exopolysaccharide	Absolute ethanol precipitation.	Sevag method; DEAE-52 column; Dialysis; Sephadex G-150 column.	Anti-inflammatory activity	[101]
	*Trametes versicolor*	Fermentation broth	Tramesan (Exopolysaccharide)	Precipitation with ethanol; Treatment with pronase E (16 h at 37 °C).	Dialysis; Low pressure size exclusion chromatography on a Sephacryl S-300 column.	Antioxidant activity	[102]
	*Tremella fuciformis*	Fermentation broth	Exopolysaccharides	Absolute ethanol precipitation	nd	nd	[103]
	*Xylaria nigripes*	Fermentation broth	Polysaccharide	Gradient ethanol precipitation (40%, 50% and 80%, 4 °C, overnight)	nd	Antioxidant activity	[104]

nd—not determined.

**Table 2:** Polyphenols and related compounds obtained from mushroom by-products.

Production Technique	Species	By-Products	Bioactive Compound	Extraction Method	Bioactivities	Reference
Submerged Liquid Fermentation	*Agaricus bisporus*	Mycelia	Gallic, *p*-coumaric, cinnamic and *p*-hydroxybenzoic acid	Extraction with methanol:water (80:20, *v*/*v*) at −20 °C for 1.5 h.	Antioxidant	[107]
		mycelia and culture media	*p*-Hydroxybenzoic, *p*-coumaric and cinnamic acid	Extraction with methanol at 25 °C, 150 rpm for 1 h.	Anti-inflammatory Cytotoxic Antioxidant	[108]
	*Inonotus obliquus*	Fermentation Broth, Mycelia	Gallic, ferulic, Epigallocatechin gallate, Phelligridin G, Davallialactone and Inoscavin B	Fermentation Broth -Extraction with 95% ethanol (1:4, *v*/*v*) at 4 °C overnight, Supernatant re-extracted with ethyl acetate (1:3, *v*/*v*) thrice Mycelial-Extraction by ultrasonication for 10 min in 70% aqueous acetone (*v*/*v*).	Antioxidant	[109]
	*I. obliquus*	Fermentation Broth, Mycelia	Epicatechin-3-gallate, epigallocatechin-3-gallate, phelligridin G, davallialactone and inoscavin B	Fermentation Broth -Extraction with 95% ethanol (1:4, *v*/*v*) at 4 °C overnight, Supernatant re-extracted with ethyl acetate and *n*-butanol (1:3, *v*/*v*) thrice Mycelial-Extraction by ultrasonication for 10 min in 70% aqueous acetone (*v*/*v*).	DPPH radical scavenging	[110]
	*I. obliquus*	Mycelia	Kaempferol, quercetin, isorhamnetin, luteolin, naringenin, apigenin, fortuneletin	Mycelial biomass was dried in a microwave oven for 10 min, extracted with 95% ethanol and stored at 4 °C overnight Centrifugation at 4500× *g* for 10 min.	Superoxide and DPPH radical Scavenging	[111]
	*Lentinula edodes*	Mycelia	Protocatechuic and *p*-hydroxybenzoic acid	Extracted with methanol:water (80:20, *v*/*v*) at −20 °C for 1.5 h.	Antioxidant	[107]
	*Pleurotus eryngii*	Mycelia and culture media	*p*-Hydroxybenzoic and cinnamic acid	Extracted with methanol at 25 °C, 150 rpm for 1 h.	Anti-inflammatory Cytotoxic Antioxidant	[112]
	*Pleurotus* *ostreatoroseus*	Mycelia	Cinnamic acid	Extracted with ethanol (70%) at 25 °C and at 130 rpm for 3 h.	Antioxidant Anti-inflammatory Antimicrobial Hepatotoxicity	[113]
	*Suillus belinii*	Mycelia and culture media	*p*-Hydroxybenzoic acid, Cinnamic acid	Extracted with methanol at 25 °C, 150 rpm for 1 h.	Anti-inflammatory Cytotoxic Antioxidant activity	[112]
Solid Substrate Fermentation	*Cordyceps militaris*	Dried powder	Shikmic, chlorogenic, syringic, *p*-coumaric and ferulic acid, rutin, genistin, daidzein, luteolin, genistein, Biochanin A	Extraction with methanol (80%) and ethanol (80%) at 50 °C for 4 h. Refluxing at 40 °C, at 150 rpm for 4 h. Centrifuged at 15,000× *g*, for 15 min at 4 °C	Antioxidant activities Inhibition of oxidative DNA damage	[114]
	*Pleurotus eryngii*	Substrate	Chlorogenic, syringic, ferulic, *p*-coumaric, caffeic, t-cinnamic, vanillic acid and naringenin	Extraction in 80% methanol under sonication for 8 h.	Antioxidant activity	[115]
	*Pleurotus ostreatus*	Substrate	Chlorogenic, syringic, ferulic, *p*-coumaric, caffeic, t-cinnamic, vanillic acid and naringenin	Extraction in 80% methanol under sonication for 8 h.	Antioxidant activity	[115]

**Table 3:** Other bioactives and related metabolites obtained from mushroom by-products.

Part A—Triterpenes and Steroidal Compounds Obtained from Mushroom By-Products							
Production Technique	Species	By-Products	Bioactive Compound	Extraction Method	Fractionation and Purification	Bioactivity	Reference
Submerged Liquid Fermentation	*I. obliquus*	mycelia	betulin, ergosterol, cholesterol, lanosterol, stigmasterol and sitosterol	Ultrasonic extraction with methanol for 2 h.	nd	nd	[122]
	*I. obliquus*	dry matter culture broth	Ergosterol, cholesterol, lanosterol and β-sitosterol	Extracted with 80% ethanol at room temperature overnight.	nd	In vivo antioxidantAnti-cholesteremic	[123]
	*I. obliquus*	Fermentation Broth, Mycelia	(22*E*)-stigmasta-7,22,25-trien-3-yl acetate, (3β)-olean-12-en-3-yl-(4hydroxyphenyl) propanoate, ligudentatol,	Extraction with hexane, chloroform, ethyl acetate and methanol three times for 12 h at room temperature	Fractionated on a silica gel column yielding four fractions further separated using a Sephadex LH-20 column	Dipeptidyl peptidase-4 inhibitory activity	[124]
Solid Substrate Fermentation	*A. bisporus*	Fruiting body	Ergosterol	Microwave-assisted extraction with ethanol at 19.4 ± 2.9 min,132.8 ± 12.4 °C and 1.6 ± 0.5 g/L	nd	nd	[125]
	*I. obliquus*	sclerotia	lanosterol, 3β-hydroxy-lanosta-8,24-diene-21-al, inotodiol, ergosterol peroxide and trametenolic acid	Extracted three times with ethanol at 78 °C. The ethanol extract was fractionated using petroleum ether and ethyl acetate as solvents.	nd	Anti-hyperglycemic α-amylase inhibitory activity DPPH radical scavenging effect	[126]
	*I. obliquus*	sclerotia	inonotusanes A, B and C, 3β-hydroxy-25,26,27-trinorlanosta-8,22*E*-dien-24-oic acid (4), lupanes and oleanane-type triterpene	Extracted under reflux with 95% EtOH for 1 h, further extracted with petroleum ether, EtOAc and *n*-BuOH	Silica gel Column chromatography [petroleum ether-EtOAc (25:1 to 1:1) and then MeOH] to obtain different fractions	Antitumor and cytotoxicity	[127]
	*Lignosus rhinocerotis*	Mycelium and sclerotium	Ergosterol Ergosta-4,7,22-trien-3b-ol	Extraction in 80% (*v*/*v*) methanol for 3 Days	nd	Antitumor and cytotoxicity	[128]
**Part B—Enzymes obtained from mushroom by-products**							
**Mushroom Production Technique**	**Mushroom Specie**	**By-Product**	**Bioactive Compound**	**Extraction Method**		**Reference**	
Solid Substrate Fermentation	*A. bisporus*	SMS	Lignocellulose-degrading enzymes	Treatment with five different solutions: distilled water, quarter-strength Ringer solution, sodium hydroxide, hydrochloric acid and potassium phosphate buffer. Incubation at 37 °C for 1 h with shaking before being clarified by centrifugation.		[129]	
	*Flammulina velutipes*, *P. eryngii*, *Lentinula edodes*, *P. ostreatus* and *Pleurotus* sp.	SMS	Cellulose-degrading enzymes: Cellulases, β-glucosidase, dextranase, amylase and laccase	Samples of the compost (wet weight) were mixed with distilled water (1:10 *w*/*v*). Incubation at 30 °C for 1 h with shaking at 180 rpm, filtrated through gauze and centrifuged at 10000× *g* at 4 °C for 100 min.		[3]	
	*Pleurotus* spp.	SMS	Lignocellulolytic Enzymes	Extracellular enzymes were extracted using four solutions from *P. ostreatus* SMS: tap water, distilled water, 50 mM sodium citrate buffer (pH 4.5) and 50 mM sodium phosphate buffer (pH 8.0). The SMS-buffer mixtures were incubated with shaking at 200 rpm for 2, 4, 6, 8, 10 or 12 h at 4 °C or 20 °C. Each sample was filtered through miracloth (pore size: 22~25 µm) and then centrifuged at 10,000× *g* at 4 °C for 15 min.		[4]	
	*A. bisporus*, *P. eryngii*, *P. ostreatus* and *C. comatus*	SMS	Laccase	Extraction with Buffer A, which contained 0.1 M sodium acetate, 5 mM calcium chloride, 0.05% Tween80 and 1% polyvinylpolypyrrolidone, on rotary shaker (180 rpm, 25 °C) for 1 h. The aqueous suspensions were centrifuged (11,000× *g*, 30 min) and the supernatants were used		[130]	
	*Agaricus bisporus*	SMS	Laccase	The residual compost (2.0 g) was mixed with distilled water (3.0 mL). The resulting mixture was kept at 4 °C for 24 h. The solid phase was recovered and pressed manually to increase the amount of liquid phase obtained. Liquid phase was passed through a mesh (0.8 mm) and centrifuged (10,000× *g*).		[131]	
	*P. ostreatus*, *Lentinula edodes*, *Flammulina velutipes* and *Hericium erinaceum*	SMS	α-amylase, cellulase, β-glucosidase, laccase and xylanase	Suspension in 100 mL of 6 solutions: 1% (*w*/*v*) NaCl, 0.1 mol/L phosphate, 0.5% (*v*/*v*) Triton X-100, 0.1 mol/L phosphate buffer supplemented with 10% (*v*/*v*) glycerol, 0.1 mol/L phosphate buffer supplemented with 0.25% (*v*/*v*) Triton X-100, and tap water. Incubation at room temperature for 1 h and centrifugation at 10,000× *g* for 5 min. In the case of using tap water, the four SMS samples were suspended in 100 mL and blended with a blender for 3 min until the suspensions were homogeneous. Filtration and incubation at room temperature for 1 h before centrifugation.		[132]	
Submerged Liquid Fermentation	*Cordyceps militaris*	Submerged culture supernatant	Fibrinolytic enzymes	After fermentation, the broth was centrifuged at 9700× *g* for 10 min.		[133]	

nd—not determined.

**Table 4:** List of non-authorized and authorized health claims for bioactive compounds that may be present in mushrooms.

Health claims	Mushroom Specie/Compound	Health Claim	Health Effect
Non-authorized	*Ganoderma lucidum*	Stimulates the body in exhaustion	Physical well-being
	*Lentinus edodes*	Contributes to natural immunological defenses	Stimulation of immunological responses
	*Pleurotus eryngii*	Regulates physiology of pancreas and fat metabolism	Improvement of the digestion of macronutrients (e.g., carbohydrates, proteins and lipids)
	*Pleurotus ostreatus*	Antioxidants can protect from radicals which cause cell damage; antioxidants can protect cells and tissues from oxidative damage.	Protection of DNA, proteins and lipids from oxidative damage.
		Contributes to natural immunological defenses.	Immune function/immune system.
Authorized	Beta-glucan	Beta-glucans contribute to the maintenance of normal blood cholesterol levels; Consumption of beta-glucans from oats or barley as part of a meal contributes to the reduction of the blood glucose rise after that meal.	Maintenance of normal blood cholesterol concentrations. Reduction of postprandial glycemic responses.
	Chitosan	Chitosan contributes to the maintenance of normal blood cholesterol levels.	Maintenance of normal blood LDL-cholesterol concentrations.
	Plant sterols and plant stanols	Plant sterols/stanols contribute to the maintenance of normal blood cholesterol levels.	Maintenance of normal blood cholesterol concentrations.
